# Hands in the Real World

**DOI:** 10.3389/frobt.2019.00147

**Published:** 2020-01-30

**Authors:** Francesca Negrello, Hannah S. Stuart, Manuel G. Catalano

**Affiliations:** ^1^Soft Robotics for Human Cooperation and Rehabilitation, Italian Institute of Technology (IIT), Genova, Italy; ^2^Embodied Dexterity Group, Department of Mechanical Engineering, University of California at Berkeley, Berkeley, CA, United States

**Keywords:** hands, field robotics, dexterous manipulation, design, mechanisms, sensing, control, benchmarking

## Abstract

Robots face a rapidly expanding range of potential applications beyond controlled environments, from remote exploration and search-and-rescue to household assistance and agriculture. The focus of physical interaction is typically delegated to end-effectors—fixtures, grippers or hands—as these machines perform manual tasks. Yet, effective deployment of versatile robot hands in the real world is still limited to few examples, despite decades of dedicated research. In this paper we review hands that found application in the field, aiming to discuss open challenges with more articulated designs, discussing novel trends and perspectives. We hope to encourage swift development of capable robotic hands for long-term use in varied real world settings. The first part of the paper centers around progress in artificial hand design, identifying key functions for a variety of environments. The final part focuses on the overall trends in hand mechanics, sensors and control, and how performance and resiliency are qualified for real world deployment.

## 1. Introduction

The human hand's ability to interact with the world for crafting, exploring and even convey emotions is one defining human characteristic that has inspired scientists and inventors for centuries. The historical emphasis on understanding the human multi-finger hand form and function likely comes from a combination of: (1) a desire to create aesthetically pleasing humanoid replicas, (2) a desire to mimic the versatility of the human hand, (3) the ease with which one can observe human hands throughout our lives, and (4) the need to perform in environments and with objects that are designed for human use (e.g., door knobs, handles, etc.). A rich record of assessing human manipulation capabilities has thus emerged, such as taxonomic grasp classifications (Cutkosky, 1989; Feix et al., 2016) and the characterization of grasp synergies during tasks of daily living (Santello et al., 1998). The human hand demonstrates great resiliency and adaptability when challenged in real world environments (Jones and Lederman, 2006).

Despite this deepening understand of human hand function, demonstrating dependable capabilities with articulated mechanized hands in real world conditions remains an open challenge; tasks, such as handling objects, can quickly become difficult due to the presence of unknowns faced in unstructured applications and environments (EU Robotics, 2016). The trade-off between task flexibility and realistic usability of manipulation systems has influenced and vexed hand designers, leading either to specific task-oriented end-effectors or to the development of fully-actuated, high degree-of-freedom (DOF) hands capable of directly-articulated versatility. Historically, single-purpose robotic end-effectors had a tremendous impact on industrial applications, being the most suitable tool for fast and highly repetitive tasks (Monkman et al., 2007). The limited versatility of industrial grippers does not fit the need of flexible and lean automation (industry 4.0) and motivates the efforts toward the development of more adaptable end-effectors, able to perform more functions than simply fixturing known objects (Fantoni et al., 2014). Unfortunately today, fully-actuated high DOF hands are commonly considered mechanically fragile and complex to control in unstructured environments; they are most suited to predetermined settings, like a laboratory. This limitation also motivates a paradigm where robotic agents in the field should avoid contact whenever possible in order to prevent accidents. This is mismatched with what we see in nature, where highly articulated animals physically interact with the world a great deal and with ease (Mason, 2018).

### 1.1. The “Real World” Design Challenge

In order to assess the current level of robotic hand development, both at technological and deployment levels, we use the Technological Readiness Level scale (TRL). TRL is one useful way to differentiate between validated investigational devices (TRL 5) and market available technologies (TRL 9), with intermediate sub-classification resolution. Today, articulated robotic hands are not broadly available in many potential markets. In this paper, we investigate applications where hands are currently at TRL 6 to 8, as found in academic literature. These application areas involve end-effectors at pre-commercial stages, that are beyond the simple proof of concept phase. Bringing these technologies to a TRL 9 level implies an additional ability to withstand the complex, application-specific demands of “real world” deployment. Real world hands are asked to perform many functions and tasks reliably, in the face of unpredictable conditions.

**Figure 1:**
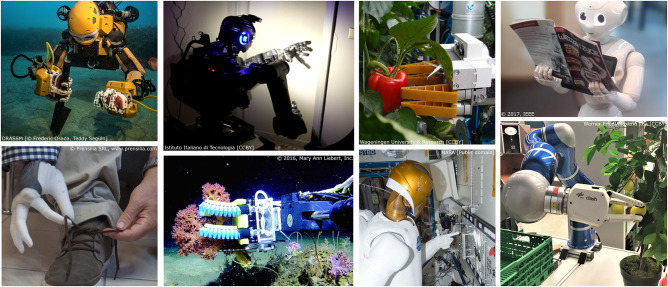
Examples of real world applied hands (Lovchik and Diftler, 1999; Hemming et al., 2014; Controzzi et al., 2016; Galloway et al., 2016; Gardecki and Podpora, 2017; Stuart et al., 2017; Friedl et al., 2018; Negrello et al., 2018). This paper focuses on field exploration, industrial applications, service robots, search and rescue and prosthetics.

This paper examines a number of relevant applications of robotic intervention, from field robotics to home settings, summarized in Figure 1. We split applications into the categories of exploring and perform maintenance in space, accessing the ocean, responding at disaster sites, performing industrial and logistical tasks, upper-extremity prosthetics and service robots capable of improving people's lives. In all of these cases, a large portion of the manual tasks required of the robot involves physical interaction with the world delegated largely to the hands.

**Figure 2:**
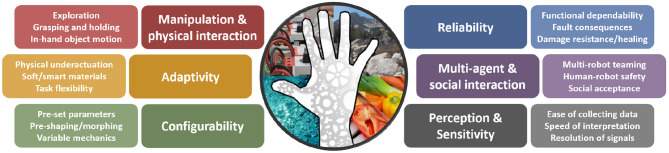
Real world hand capabilities, including key elements for the function or behavior of the device during real world operations.

Hands are often expected to grasp a wide range of objects of varying geometry and mechanics (e.g., shape, friction, softness, etc.) or manipulate tools in many different ways (e.g., twisting, pushing, wrenching, tearing, etc.). A number of works address the kinematic and dynamic ability of a hand to perform these desired capabilities (e.g., Mishra et al., 1987; Montana, 1988; Li et al., 1989; Murray, 2017). In addition, as end-effectors are extended away from a central body, at the ends of arms, hands often make contact with the world, whether intentional or accidental; they are subject to impacts and extreme loading, and thus require a high degree of physical resiliency. For hands to be successfully deployed in the real world, designers must meaningfully address all aspects, which often requires trade-off between task flexibility, mechanical robustness and others application constraints, like cost, size and weight. Figure 2 provides an overview of the main capabilities and functional requirements that are relevant for real world hands and that are discussed in this work.

Among state of art hands, adaptive appendages are proving to be a useful solution, affording passive physical robustness while enabling a wide range of behaviors. Recent trends toward soft, multi-material and sensitive designs show growing importance when researchers work toward robots in more unstructured tasks. Yet, these devices create fundamental challenges in the areas of control and artificial intelligence. Small changes in the embodied agent—the *mechanics* of forces and motions and *sensors* that provide information for situational awareness—can have dramatic outcomes for the algorithms designed to command the hand. The future of hands requires a tighter interconnection between hardware, sensing and control, especially for challenging unstructured environments.

### 1.2. Overview

Predominant applications of hands are briefly reviewed in section 2 and the primary considerations observed for each real world deployment are qualitatively identified. A perspective on the open challenges and novel trends is then discussed in section 3, aiming to encourage swift development of more versatile real-world-capable articulated robotic hands. Special focus is given to the duality between dexterous function and realistic adoptability in unforgiving environments, and trends in benchmarking these properties. The goal is to discuss and provide insights that, in the opinion of the authors, should help to bridge the gap between academic investigations and robot application.

## 2. Hands in the Field

**Figure 3:**
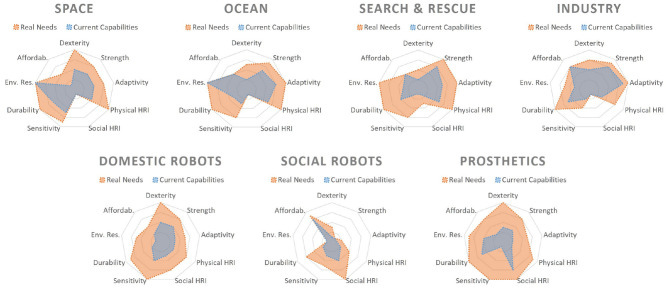
A graphical representation of the authors' qualitative opinion. A score of 1–5 indicates the estimated state of technological development of the hands in different fields. The blue layer (current capabilities) it is based on well-known examples of application retrieved in literature (e.g., Chalon et al., 2011; Gardecki and Podpora, 2017; Stuart et al., 2017; Negrello et al., 2018; Honda, 2019; Ottobock, 2019a; SHUNK Robotics, 2019), while the orange layer is a qualitative representation of the opinion of the authors based on the review reported in section 2.

This section provides an overview on the hands that have been deployed in the real world, highlighting the different device requirements and discussing their main characteristics regarding hardware, control, and sensing (Figure 3). In each section are reported selected notable citations or fields of uses for reference. On whole, hands for each application area are constrained by consumer expectations and the physical properties of the environment, including the objects that must be dexterously manipulated.

### 2.1. Space

Space operations are classified as intra-vehicular activities (IVA) or extra-vehicular activities (EVA), and dexterous robots are suitable for both. Automated assistants could be applied during relatively standard and repetitive IVA (e.g., push buttons, open drawers, etc.). The inside of space capsules are designed for human use, so it may be expected that the robot can handle human-relevant tools and objects. EVA can be dangerous for astronauts; space suits make movements more clumsy and accidents can result is loss of life (Hirzinger et al., 2003). This motivates the development of robotic solutions capable of more versatile tasks in open space. Limited thermal dissipation in space leads to motor overheating, which can severely affects hand operation duration. While heat can be controlled with periodic shut down, particular care should be devoted to the design of electronic board, especially when they need to be continuously operated (Chalon et al., 2011).

Various efforts have sought to develop adaptive and reconfigurable hands for these environments, such as in works by Lovchik and Diftler (1999), Laliberté and Gosselin (2001), and Chalon et al. (2011). Despite the relevance of the application, few hands for space operations have been deployed, due in part to extremely strict technical requirements and the high cost of testing. Both during IVA and EVA, the robot may be working alongside people in an interactive fashion, and unintended damage to the vessel could compromise crew safety and mission success. It is considered mandatory that hands ensure safe physical interaction with humans and the environment at all times. This is one reason why hands designed for space universally include embed tactile or force sensing in the fingers.

It should be noted that, on Earth's surface, designers must carefully reduce end-effector mass, which disproportionately influences manipulator payload and inertia. While in space, the mass of the hand may be considered less critical to the performance of the system. However, severe weight constraints may remain due to launching restrictions. The handling of floating objects, which are much easier to push away from the hand during grasping, additionally creates new challenges is design, sensing and control unique to space applications.

### 2.2. Ocean

Marine manipulation operations are dominated by industrial applications, e.g., equipment maintenance, or scientific exploration, e.g., collecting geological cores. Ocean devices must be able to survive large changes in ambient pressure associated with changing depth, and prevent water from getting into and damaging vulnerable materials, like electronics. These hands therefore either tend to minimize the complexity of the mechanism with reduced numbers of actuators, seals, joints and sensors. Devices intended for repeated use include especially reliable waterproofing and specialized corrosion-resistant materials.

A number of hands are deployed in the ocean, from single-DOF rigid claws, such as the Schilling Titan gripper^1^, to adaptive grippers with compliant elements or underactuation (e.g., Cianchetti et al., 2011; Lemburg et al., 2011; Bemfica et al., 2014; Galloway et al., 2016; Laschi, 2017; Stuart et al., 2017; Mura et al., 2018; Takeuchi et al., 2018; Sinatra et al., 2019). While the non-adaptive end-effectors are most consistently utilized, they are not as suitable for nondestructive, gentle biological and archaeological tasks. Though rare, due to implementation challenges, some groups have included tactile sensing to undersea hands, choosing modalities or designs that make the reading insensitive to changes in ambient pressure or cold temperatures (e.g., Lane et al., 1999; Dennerlein et al., 2000; Sanz et al., 2013; Aggarwal et al., 2015; Kampmann and Kirchner, 2015).

There are numerous undersea gripping solutions use fluid pumps. For example, the universal jamming gripper modified for underwater purposes can handle irregularly shaped objects in submerged conditions (Licht et al., 2016). Hydraulic actuation makes sense under water, as it will be approximately neutrally buoyant. Water is a denser and more viscous fluid as compared with air, so direct suction attachment is an especially attractive mechanism. Limpet-inspired suction cups have been applied for artifact gathering in the deep-sea, such as at shipwreck sites (Søreide, 2011). Suction flow was incorporated onto a multi-finger underwater hand (Stuart et al., 2019), and the monitoring of suction flows has been introduced as a potential way to perform tactile sensing under water (Stuart et al., 2015). Even without suction pumps, water drag effects on objects has been shown to improve the capture of floating objects, as compared to grasping in the vacuum of space (Stuart et al., 2019).

### 2.3. Search and Rescue and Disaster Scenarios

Disaster response activities are dominated by intense workload, e.g., for using high power tools or for removing debris. At these large force applications, reinforced mechanisms, physical interlocking with the object and high-friction contacts are deemed critical. Recent works to grip onto rocky terrain even includes spikes and spines to increase contact forces (Ruotolo et al., 2019; Wang et al., 2019). Difficult terrain leads to agent falls and collisions, such that the hand will need to support body weight during whole-body locomotion (Negrello et al., 2018). The DARPA Robotics Challenge (DRC) provides an example of different activities performed by disaster responders (DARPA, 2019). Concerning manipulation, most of DRC tasks required wrist dexterity rather than finger dexterity, therefore, many teams opted for simplified hands or grippers (Stentz et al., 2015; Karumanchi et al., 2017; Tsagarakis et al., 2017), and sometimes even just hooks (Johnson et al., 2015). This choice was justified by the need for robust and reliable end-effectors prioritized over dexterous hands.

Among different implementations, it is worth mentioning that the most used solution by DRC teams was the Robotiq 3 fingers gripper (Robotiq, 2019) which, thanks to its underactuated fingers, provides an adaptive grasp. Similarly, several teams leveraged underactuation and compliance to develop end-effectors easy to use (Rouleau and Hong, 2014) and durable, thanks to the small number of components (Stentz et al., 2015; Negrello et al., 2018). Another benefit of underactuation is the possibility to locate sensing systems away from the fingers, thus reducing the number of electrical components that could potentially be damaged during physical interactions (Catalano et al., 2014).

This field application is rapidly evolving. Although at a very early development stage, it is worth mentioning the advances in aerial manipulation for search and rescue and maintenance activities at high altitude. In this emerging field, the development of ultra-lightweight manipulators and end-effectors are emphasized, as they directly change the payload and battery life Ruggiero et al. (2018). One engineering step that is currently missing is the capability to withstand wet conditions and high temperature changes which are common in disaster scenarios (e.g., firefighters).

### 2.4. Industry and Logistics

**Figure 4:**
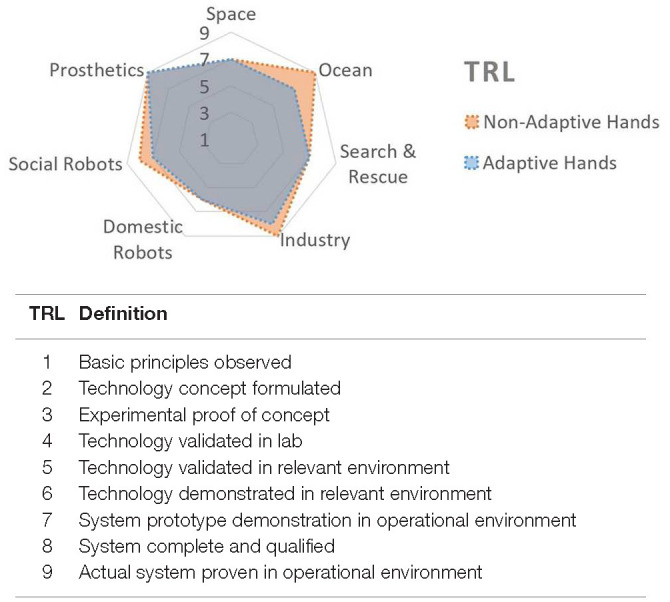
A comparison of the current technology readiness level (TRL) over different application fields, based on the authors' qualitative opinion **(Top)** and the definition of the TRL provided by the European commission **(Bottom)**. Available online at: https://ec.europa.eu/research/participants/data/ref/h2020/other/wp/2018-2020/annexes/h2020-wp1820-annex-g-trl_en.pdf

Grippers for industry are one of the most mature technologies of this review, being successfully employed in industry in the last 50 years (TRL 9, Figure 4). This application has multiple sub-domains, such as logistics, assembly lines, waste management and agrifood (Fantoni et al., 2014). Typical tasks, such as in warehouses and production lines, require robotic systems that are cheap, robust, easy to control, and capable to reliably grasping a large variety of objects (Kragten et al., 2012; Asfour et al., 2018; SHUNK Robotics, 2019). For this reason in the last years many companies have exploited pneumatic actuation for the development of soft continuous grippers (Soft Robotics Company, 2019) or non-conventional granular grippers (Brown et al., 2010; Amend et al., 2016). Simplicity in integration and use is prioritized for this robotic equipment such that it can be employed by non-expert users (Franka Emika, 2019; Robotiq, 2019).

Performance is typically described in terms of long-term durability and speed of picking objects. Speed is typically referenced in terms of human operator capabilities: 5–10 s/item (Amazon Robotics, 2019). Other requirements depend on the specific application, e.g., in agrifood field it is necessary to handle fruits and vegetables gently to prevent damaging goods. In this case, we observe a trend in developing soft robotic systems to provide both adaptiveness and delicacy (Deimel and Brock, 2013; Deimel and Brock, 2016; Friedl et al., 2018; CROPS Consortium, 2019). In this type of sub-domain, sterilizable end-effectors help reduce the risks of contamination (e.g., food industry, clean room).

Another relevant application is autonomous maintenance in remote (power plants, offshore platforms) or dangerous sites (nuclear reactor, particle accelerators, tanks and vessels) (Parker and Draper, 2007). In these environments, hands are usually expected to perform relatively simple manipulation tasks, like turning valves, or retrieve items (CERN, 2019). An emerging application is waste management, such as domestic (Zen Robotics, 2019), nuclear and hazardous scums (ROMANS European Project, 2019), including decommissioning. Again, sterilization of the hand is essential, for decontamination after handling hazardous materials or operations in hazardous areas (SHUNK Robotics, 2019). In maintenance, one challenge is related to the problem of sorting a large variety of shapes and sizes, including heavy and bulky materials.

### 2.5. Service Robotics

Many robots with very different characteristics belong to this category. Therefore, it is worth distinguish between those which are meant to perform significant physical work, e.g., helping elders, from those meant only for entertaining or social interaction purposes.

*Domestic robots* meant to perform manual labor, possess a relatively high level of dexterity and strength, e.g., for the handling cooking tools and to perform duties usually performed by humans (Asfour et al., 2008; Wang et al., 2008). Among other characteristics, it is important that these hands are affordable for adoption, and resistant to soft collisions (Sureshbabu et al., 2017; Honda, 2019). Despite these robots having been introduced in the collective imaginary by Asimov's books, still this field is an open technical challenge for researchers and there are no humanoids robots deployed reliably in homes yet.

With robots designed primarily for *social interaction*, it is particularly relevant how they behave and if they hold human-likeness, which is defined by the well known problem of the uncanny valley (Mori et al., 2012). In this context, hands have a crucial role for humanoid robots since they provide them the capability of expressing, gesticulating and conveying feelings (Fong et al., 2003). Most of these robots possess hands characterized by simple design and basic grasp capabilities (Kaneko et al., 2009; Gardecki and Podpora, 2017; Pal Robotics, 2019). Integration of sensors for detecting contacts on arms and palms is relevant (Gardecki and Podpora, 2017). In the research literature, there are some examples of specially developed sensors for touch Schmitz et al. (2010) and hugs (Alspach et al., 2018). The overall acceptance of social robotic systems is strongly dependent on the cultural background of people and nations and these differences should be taken into account (Lee et al., 2016).

### 2.6. Prosthetics

Prosthetic hand technologies are some of the oldest and most mature among the others presented in this work (Figure 4). Most of the products on the market are 1 DOF systems, being more similar to a simple gripper than to a hand. Prosthetics can be classified as cosmetic hands, body powered (BPPs) or myo-electric (Kulkarni and Uddanwadiker, 2015). BPPs are probably the most used thanks to their robustness, ease of use and low cost. In the last 20 years new hands with a higher level of articulation have been developed in an attempt to bridge the gap between the artificial and human hand kinematics (Ottobock, 2019a,b; Touch Bionics, 2019). Despite these efforts, existing devices, especially the ones with multiple degrees of freedom, are difficult to control intuitively, and in some cases are bulky and fragile; they remain far from the vision of seamless human-machine interaction or providing capabilities identical to the natural counterpart (Chadwell et al., 2016; Cordella et al., 2016). On whole, hand prosthetics still lacking in one or more of the following performance: functionality, durability, cosmetic appearance, and affordability (Cordella et al., 2016). Consequently, about 20% of amputees tend to abandon the use of their upper limb prostheses (Biddiss and Chau, 2007).

The addition of adaptivity and sensitivity to prosthetic devices is growing in scientific popularity. Recently, technological solutions and scientific findings are giving rise to a generation of prosthetic hands characterized by an anthropomorphic architecture (i.e., multi-fingered, multiple degree of freedom), but with a reduced number of degrees of actuation, with the aim to establish balance between functionality, mechatronic complexity and easiness of use. Results from these efforts are promising (Piazza et al., 2019), however few of them are used and tested in realistic contexts (Godfrey et al., 2018). Robotic prosthetics on the market are relatively mature, however they do not yet provide rich forms of feedback to the user, such as active haptics. This issue is now considered a paramount need Lewis et al. (2012), and is an active area of research, for example in works such as in Antfolk et al. (2013), Kim and Colgate (2012), and Battaglia et al. (2017).

Another important requirement for these systems is energy efficiency. One example is provided by prosthetic hands that include non-backdrivable actuator transmissions to avoid to dissipate energy while holding an object and reduce end-effector weight (Montagnani et al., 2015b). This is a solution utilized in various hands that must provide strength even when the motors are powered off.

## 3. Discussion and Perspectives

Section 2 demonstrated the breadth of real world applications suitable for robot hands and manipulation. Physical resiliency is one of the key aspects observed over different real world applications. Simple yet robust end-effectors tend to be preferred with respect to more complex and potentially unreliable ones. However, a variety of different abilities are demanded to satisfy the requirements of real world deployment (see Figure 2). Figure 3 shows the current state of-the-art in the development and use over the different application fields. For each ability, a qualitative value based on author opinion, supported by select examples found in literature, is assigned to demonstrate the state of hands used in real world applications. Figure 4 shows the author's qualitative perception of current technology readiness levels (TRL). On the basis of the collected results and observations, this section aims to provide a discussion on novel trends in robotic hands design, sensing and controls and a perspective on what is missing toward larger deployment of articulated hands in real world.

### 3.1. Trends in Hand Mechanics

**Figure 5:**
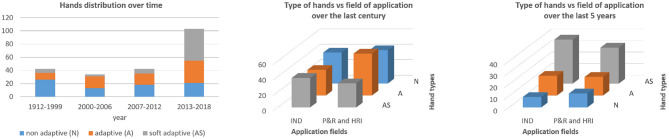
Data extracted from the database provided in Piazza et al. (2019), **(left)** shows the hand layout distribution over the years, **(middle)** and **(right)** show the hand applications (industrial, prosthetic and human robot interaction) over the full century and the last 5 years, respectively. The database is available at the link: https://www.annualreviews.org/doi/suppl/10.1146/annurev-control-060117-105003.

Until recently, the most common solution was the single degree of freedom claw or gripper. They are resilient, simple and capable of a variety of mission-critical tasks. Yet, they are specialized for interacting with tools, structures, and resilient materials. More articulated, fully-actuated hands enable task flexibility and adaptive mechanisms introduce the potential to evenly distribute contact forces passively when interacting with objects for more gentle handling of objects (Birglen et al., 2007). Yet adaptive grippers tend to trade off precision, conformability and task flexibility. The first graph of Figure 5 shows the number of hands developed over the last century (data extracted from the database provided by Piazza et al., 2019), divided by non-adaptive hands (N) included fully actuated and coupled solutions, adaptive hands (A) consisting of underactuated and compliant transmissions and soft adaptive hands (AS), implemented with an adaptive transmission and soft materials. The second graph of Figure 5 shows the distribution of hand designs over different fields. It is worth noticing that the industrial field (IND) hands come more evenly across a variety of solutions, including AS, while in the prosthetic (P&R) and human robot interaction designs (HRI) utilize more traditional mechanisms (N & A). In the last 5 years there is an impressive number of hands that combine underactuation and compliance, as shown in Figure 5. This may be driven by new robotic applications in highly unstructured environments.

Soft robotics can include both rigid links and elasticity materials (e.g., flexible joints Stuart et al., 2017) or those made entirely of compliant materials (e.g., soft continuous robots Deimel and Brock, 2013). Fully-soft hands use a wide variety of actuation principles. Pneumatic actuation today has an increasing appeal, despite the traditional limitation related to the difficulty of integrating a power source on a robot for long lasting untethered applications. Soft continuous robots offer broad possibilities, such as adaptability, squeezability, and even morphing or evolving structures, as discussed in Laschi et al. (2016), where researchers envision hybrid soft-rigid systems to overcome some of the limitations of traditional polymeric structure (e.g., max actuation force) to further increase their application outside of the lab. Although at a preliminary stage, research on new polymeric materials may have a huge impact on future field application, allowing to develop self-healing (Shepherd et al., 2013; Terryn et al., 2017), and biodegradable robots (Rossiter et al., 2016). Within the flexible joint category exists a variety of implementations including flexures and dislocatable joints (Catalano et al., 2014), which allow large motion between two phalanxes (disarticulation). For a deeper classification of hands joint design and their applications we refer to Piazza et al. (2019), where a complete review of robotic hands over the last century is reported.

Currently, the emerging trend in reduction of mechatronic complexity and degrees of actuation is generating solutions with high grasping performance, but, apparently, reduced capabilities in term of dexterity. Such situations are rapidly changing thanks to the introduction of novel control paradigms (for more details see section 3.3) or design solutions were a balance between complexity and dexterity is achieved trough the combination of different technologies (Tincani et al., 2013; Spiers et al., 2018) or actuation architectures (Alspach et al., 2018; Della Santina et al., 2018).

Humanoid hands have emerged in all application areas, either for aesthetic or functional purposes. It is worth discussing how finger functionality in human hands has been adopted in the robotic domain. Observing hands in literature, it seems there is no single agreement on the morphology of anthropomorphic hand both in shape (# of fingers) and in size. However, the need for opposing fingers, like a thumb, is recognized both in anthropomorphic and non-anthropomorphic end-effectors alike (Lin and Sun, 2014; Mason, 2018). At the same time, human hand morphology is not consistently defined, presenting large variations in size and kinematics over the world population (Grebenstein et al., 2010). For example, some people affected by polydactile syndrome are equipped with six fingers; recent studies demonstrate how this extra digit enhances the dexterity of these individuals (Mehring et al., 2019). There is also evidence from hand reconstruction surgical literature that the various fingers of the human hold different specializations based on their position on the palm (Zenn and Levin, 2005), such that the person's occupation must be considered when prioritizing different digits. As noted in Cutkosky and Howe (1990), established theories regarding virtual fingers can assist in understanding the collective role of multiple fingers acting together to perform dexterous manipulation and grasping. These types of studies into human hand function are indeed paving the way to new dexterous hands designs in the future.

While this article addresses multi-finger hands, we acknowledge that end-effector effectiveness is influenced by the motions of the more proximal joints. For example, human wrist motions play a critical role for reaching and fine adjustments of a grasp (Ma and Feldman, 1995; Montagnani et al., 2015a). Arm action will especially influence highly underactuated and compliant grippers. Thus, as hands continue to trend toward adaptivity, the creation of complementary robotic wrists is becoming more and more significant in grasping and manipulation (Bajaj et al., 2015; Casini et al., 2017; Negrello et al., 2019b).

### 3.2. Sensitivity

Sensing the environment is critical to enabling grasping and manipulation that responds effectively in unstructured environments. A rich set of recent reviews and chapters tackle this broad and important field of research for hands, such as Kappassov et al. (2015), Yousef et al. (2011), Cutkosky et al. (2008), and Tegin and Wikander (2005). In grasping, as hands continue to trend toward more adaptivity and underactuation, the demand on collocating sensors at critical contact locations increases. These hands are highly influences by contact conditions, which are notoriously very difficult to model accurately or identify with sight alone. Thus, we focus on the development and interpretations of tactile information.

Some artificial skin designs for hands can provide high spacial contact resolution, in an attempt to approach the dense array of tactile sensors found on the human fingertip (e.g., Johansson and Flanagan, 2009). However, resolution is only one way to assess the effectiveness of skin sensors. Bandwidth, sensitivity and range will all affect the usefulness of a sensor in different circumstances. For example, during a fast impact with a blunt object, bandwidth and range may be prioritized over fine spacial resolution or sensitivity. As robots face diverse sets of challenges in the real world, it becomes difficult to define clear performance metrics that will enable a hand to react resiliently and effectively given unexpected stimuli. Researchers have looked to nature to find inspiration for incorporating and interpreting tactile information (e.g., Romano et al., 2011). Other groups look to machine learning methods to perform tasks, for example haptic SLAM (EU Robotics, 2016).

Current tactile sensing technologies are highly variable, utilizing a wide range of physical phenomenon to, most often, estimate contact forces. The most common solutions use pressure transducers, capacitive plates, light reflectivity, etc. to measure skin deformations given external forces. Some focus on dynamic signals, i.e., cannot measure steady state forces, with sensors like PVDF that only respond during a change in stimuli. Of course, there are groups who explore other physical modalities to measure contact conditions, such as temperature gradient as a way to detect slip in specialized tasks (Burkhard et al., 2017). There are practical issues when including sensors at distal locations on flexible appendages. Wires flex and bend as the hand opens and closes, resulting in cyclical straining which tends to make tactile sensors connections break. New methods to create stretchable, flexible or high bandwidth wireless sensors will continue to make more rich sensing possible at the contacts of real world end-effectors. Efforts that utilize sensor fusion (e.g., tactile and visual sensing) will additionally allow multiple different sensing modalities to create a richer sense of physical interactions with fewer collocated taxels.

### 3.3. Control and Planning

The control of multi-fingered hands has historically (since the 1960's) addressed the grasp planning process. The fingers, when performing a grasp, must interact only with the object, without perturbing the equilibrium of the object, the environment, or the hand itself. Proposed methods following this paradigm are, for example, the kinematics and/or dynamics driven approaches, where, in order to obtain a stable grasp, the exact computation of the fingertip positions are required (Bicchi and Kumar, 2000), or the one introduced by Ferrari and Canny (1992) which, to obtain an optimal force closure grasp, considers the total and maximum forces exerted by fingers. Such approaches, developed as a direct consequence of the rigidity of fingers and the intrinsic fragility of the hands, rely on precise knowledge of the object and of the environment surrounding it. Simulations of such systems usually require large computations (Bohg et al., 2014) and careful consideration of contact condition modeling. More recent approaches rely on grasping data-sets, usually obtained from trials on real robots, or from simulations. Experimental strategies are more stable than the model-based ones and are capable of capturing perceptual uncertainties and execution errors, commonly present in real circumstances (Bohg et al., 2014).

Although of great relevance, many of the above solutions do not match behaviors and strategies adopted by humans. Indeed, by observing how people manipulate objects, it possible to observe how the inherent compliance of the human hand plays a significant role in grasping actions (Bonilla et al., 2014; Eppner et al., 2015). Such considerations, together with the emerging trend of novel soft and under-actuated hands (Piazza et al., 2019), is opening innovative opportunities and challenges regarding the control and planning problem of robotic hands.

The way that compliant and underactuated hands behave depends on the physical interaction, i.e., the shape, mechanics and surface properties of the grasped object. This is in contrast to fully-actuated hands, where detailed finger motions can be planned and executed, with contact force commanded for each independent fingertip. One result is that under-actuation can simplify control, as the embodied intelligence of the mechanism can passively couple motions necessary for tasks like wrap grasping. In the case of soft grasping a large number of simple problems can be addressed with a minimal amount of visual information and a simplified grasp planning (Krahn et al., 2017; Al-Ibadi et al., 2018). The final resting posture of each finger and the force at each contact depends on a balance of tendon forces, joint stiffnesses, and contact friction forces. However, the uncertainties linked to the continuous balance among many grasping parameters leads to reduced control authority. This is why these hands are typically used for grasping and holding objects, and less so to perform dexterous maneuvers (although a few specific in-hand manipulations can be possible, for example picking up a small object Odhner et al., 2013; Godfrey et al., 2018 or switching from a pinch to a wrap grasp Aukes et al., 2014; Della Santina et al., 2018). Consequently, this review does not discuss deeply in-hand manipulation challenges, although it has been an open research question for at least the last 20 years (Okamura et al., 2000; Nagabandi et al., 2019).

Using hardware which can safely and resiliently contact the environment a great deal without negative consequences, e.g., when compliance is included, leads to new possibilities, such as exploratory touch and exploiting the environment for achieving new grasps. These methods help to overcome the more narrow grasping primitives used as kinematic reference during the design phase of the hand (Bonilla et al., 2014). Such opportunities can emerge from both the observation of humans performing actions with robotic hands and data driven methods (Bonilla et al., 2014; Pacchierotti et al., 2014; Della Santina et al., 2019). Enabling touch sensing capabilities additionally creates new avenues of control, playing, in such a way, an important role as in humans.

### 3.4. Resiliency

As previously discussed, the cost of failure can dramatically vary between different real world applications. For example, during remote mobile exploration in the ocean or in space, where the cost of operation is immense, functional failure is unacceptable. However, for a home assistive device that can more easily be serviced, failure may not seem as catastrophic. Therefore, each specific application will have different demands on performance and lifetime, depending on the customer. We use the term *resiliency* to broadly capture a hand's ability to act with functional robustness. While this includes simple physical sturdiness, there may be other aspects such as redundancy. System resilience, in its broadest definition, refers to the capability of absorbing damage without a complete function loss (Yodo and Wang, 2016). This includes the concepts of dependability, which is the capability to repeat a task or a performance appropriately even in presence of failures, and durability and reliability, as the ability of a system, or component, to work under certain conditions for a specified amount of time (EU Robotics, 2016).

Hands should be able to cope with task uncertainties and unpredictable interactions. This could be a hand's ability to perform with significant sensor or computational error, or a hand's ability to demonstrate excaptation, or flexibility to perform tasks that are not originally intended. In this sense, a resilient system, not only should be characterized by high damage resistance, but also should take into account changes in the system itself (reconfigurability). Elements that we consider critical for the development of resilient systems that could effectively operate in real world involve *hardware* and *sensing and control*.

On one side, during the design of *hardware*, it is crucial to enhance the physical sturdiness of those systems which have a higher failure cost, for instance by introducing compliance (Negrello et al., 2019a). On the other side, maintainability and lightness are considered essential. Among the approaches in literature, is worth mentioning modularity (Hirzinger et al., 2003), underactuation (Stentz et al., 2015; Negrello et al., 2018), and mechanical fuses with easily-replaced sacrificial components (Shaw, 1972). Affordability also has an important role. In section 2, most hands are developed directly by the users, with great financial investment and internal know-how. Currently, a few groups share designs, schemes and controllers with the community (Santina et al., 2017; OPENROBOTHARDWARE, 2019), and these solutions tend to be limited to simple fabrication methods. Hopefully, these efforts will contribute to bring on the market more cheap and reliable hardware, to foster participation and research into deploying hands outside of the lab. Preventing *sensing and controller* failure first requires integrating opportune sensing systems to monitor hand status. Then, criteria to define self-diagnosis should be developed. Finally, control systems modulate parameters given sensor feedback for self recovery and coping with failures.

For hands to be successfully deployed in real world applications, designers must meaningfully address all above aspects of resiliency, and understand the sometimes complex interconnections between the decisions regarding hardware, control and sensitivity with a system-wide perspective.

### 3.5. Benchmarking

One aspect limiting the industrial appeal to underactuated soft grippers is the inability to formally compare and contrast different designs. Benchmarking serves a dual purpose of providing researchers and developers with tools for assessing and improving their end-effectors, while supporting end-users in the selection among different products on the market.

In the last 5 years, the dexterous manipulation community has been very active in the benchmarking effort, both on the scientific and technical side. One example is the number of competitions and events issued, like the Amazon Picking Challenge (Correll et al., 2018; Amazon Robotics, 2019), or the Robotic Grasping and Manipulation Challenges (RGMC) held in conjunction with IROS 2016–2017 (Falco et al., 2016; Sun et al., 2016). On the scientific side, the efforts where devoted to the development and collection of data-sets (Calli et al., 2015) and the formalization of tests for performance evaluation of basic end-effector capabilities (Falco et al., 2015). For complex and industrial oriented tasks, works include those by Sotiropoulos et al. (2018) and Leitner et al. (2017).

A benchmark should provide clear metrics and results and be easy to replicate and use. One of the difficulties in defining a benchmark for robotic hands and manipulation is related to the strong interconnection among hardware, sensitivity and control. Here, a question arises regarding whether it is worthwhile performing the complete evaluation of a robotic system (hardware and software) or to develop tests for characterizing each individual component. As a result from the RGMC, it emerged that, while specific tests are needed for the objective evaluation of hand hardware performance, competitions can better focus evaluation on the hand manipulation capabilities at a system level, e.g., fully autonomous grasping and manipulation frameworks (Falco et al., 2016). Moreover, each specific application defines task parameters which are most relevant, and which should be prioritized when designing an end-effector, e.g., speed of picking objects should prioritized in industrial setting over dexterity or sensitivity. Accordingly, benchmarks should be application-oriented and tuned to highlight the performance as a function of focused parameters. In this respect, NIST is developing a complete framework that ranges from the unit tests up to functional tests for global manipulation system assessment (Falco et al., 2018).

As discussed in section 3.4, hardware resiliency is an unavoidable requirement for real world application, and in such contexts physical robustness holds particular importance. Based on the Izod and Charpy test for material toughness characterization, a method for assessing the robustness of artificial hands was proposed for dynamic loads such as impacts (Negrello et al., 2019a). Among the results, it is worth highlighting that compliance enables more system resiliency and reduces the transmission of loads to the robot's central structure. A more systematic application of such tests could assess hardware capacity to survive impacts and provide insights for designers, e.g., for material selection or for identifying the most suitable actuation layout.

### 3.6. Certifications

Another important market barrier is represented by certifications, which are legal requirements and rules the products must comply with (e.g., CE mark) in order to be sold within certain market segments, e.g., industry or prosthetics. Currently, very few end-effectors on the market (except industrial grippers and commercial prosthetic hands) are compliant with normative guidelines. This is not only because of high standards and tight design requirements (e.g., IP 67 or fatigue life from 500,000 to 1 million cycles), but also to the novelty of the topic (e.g., the ISO norms for collaborative end-effectors, ISO/TR 20218-1 were released in November 2018). To the best of authors knowledge, SHUNK (2019) and QB Robotics (2019) are two of the only certified hands currently available on the market. This aspect should not be underestimated for real world adoption, since it has substantial impact on company organization and production methods. Standards and certifications differ among countries and areas, making the process to certification rather clumsy.

In field activities, exposure to natural environmental conditions may introduce mechanical problems, like abrasion, clogging, blocking or corrosion and may cause electronics failure. Therefore, it is important to include in certification evaluation framework tests to guarantee field serviceability (Gould and Maciel, 1995) and safety. In the automotive sector, this aspect has been investigated since the 1960's (Nock et al., 1968), developing a rather articulated and complete set of tests for assessing product functionality in specified environments, including different temperature, rain, humidity and pressure, dust and sand conditions (SAE J1455). We envision a set of environment-specific tests for robot end-effectors, which, as the primary conduit of physical manipulation, could be subject to highly variable conditions and frequent damage.

## 4. Conclusion

Hands are an essential element for robot manipulation in real world applications, and present a multifaceted challenge; they are asked to perform physical interactions with great reliability and environmental uncertainty. In this review article, we presented recent trends regarding emerging real world applications in order to highlight and motivate continued work on open issues.

Single-purpose jaws and grippers are the historical standard for robotic manipulation outside a controlled lab setting. Thus, the majority of more articulated hands are at early prototypical or commercialization stages of development; continued work into hand design and control holds great potential to enable effective deployment of more versatile and varied solutions. Recent trends indicate growing interest in developing adaptive hands, that utilize underactuation and compliance. Such hands can improve physical resilience and ease the demand on control computation, yet also provide new challenges in fabrication, sensing and grasp planning during complex interactions. State of the art systems lack dexterity, sensitivity and resilience with respect to what is required by real world activities and is an ongoing research effort. To fill the current gap between existing prototypes and product requirement for real world deployment, researchers are developing benchmarks and hand functional characterizations, certifications and normative references.

As hands for real world operation continue to develop, it becomes increasingly apparent that this is a highly multi-disciplinary effort. Hardware system designers, electrical engineers and experts in control and artificial intelligence must interface in order to continue extracting the interdependent trade-offs among all fields. The ultimate goal is to create more capable manipulation devices, and reduce the cost of failure. Eventually, robot hands may be able to match the elegance and robust multi-functionality witnesses in the human hand.

## Author Contributions

All authors listed have made a substantial, direct and intellectual contribution to the work, and approved it for publication.

### Conflict of Interest

The authors declare that the research was conducted in the absence of any commercial or financial relationships that could be construed as a potential conflict of interest.

## Footnotes

^1^Schilling Robotics technologies, online resources. Available online at: https://www.technipfmc.com (accessed January, 2020).
